# Effects of a Psychological Management Program on Subjective Happiness, Anger Control Ability, and Gratitude among Late Adolescent Males in Korea

**DOI:** 10.3390/ijerph17082683

**Published:** 2020-04-14

**Authors:** Ju Eun Hong, Miok Kim

**Affiliations:** 1 Department of Nursing, Dongyang University, Yeongju 36040, Korea; nurse0413@dyu.ac.kr; 2 Department of Nursing, College of Nursing, Dankook University, Cheonan 31116, Korea

**Keywords:** adolescents, gratitude, emotional difficulties, anger, happiness

## Abstract

This study aimed to develop an emotion management program for late adolescent males, and investigate the effect of the program. This study is an experimental study using a pretest-posttest control group design. It was conducted from 14 May to 21 December, 2018 at a male high school in Korea. It analyzed 52 participants (26 in the experimental group and 26 in the control group.) Regarding subjective happiness, there was a significant difference between groups (t = 3.409, *p* = 0.001). In anger control ability and gratitude, there was no significant difference between groups (t = 0.332, *p* = 0.740/ z = 0.528, *p* = 0.599). This program for emotion management, which is based on the broaden-and-build theory, can improve subjective happiness, anger control ability, and gratitude, by reinforcing appreciation skills and anger awareness and self-expression in male high school students.

## 1. Introduction

Happiness is characterized by psychological well-being, subjective well-being, and life satisfaction, and these aspects are used as indicators of happiness from a quality of life perspective [1]. According to the 2014 Korean Children and Youth Happiness Index Survey, the subjective happiness index among Korean children and adolescents was 74 (out of 100). This is the lowest among 23 Organization for Economic Co-operation and Development (OECD) countries that utilize this index, and Korea was ranked at the bottom for six consecutive years, from the first survey in 2009 up to 2014 [2]. In particular, it is believed that study results, showing that high school students, who are in their late adolescence, have low happiness, are believed to reflect the academic stress brought upon by the college admission-oriented education system and competitive school atmosphere [2,3]. Some high school boys are known to have difficulties with their school lives due to stress, anxiety, fatigue, and mind-body imbalance caused by excessive school load [4].

According to the Youth Health Behavior Online Survey [5], 28.7% of high school students have experienced mental health problems, such as depression and stress, which can induce sleep disorders, delinquency, substance abuse, and other physical health problems. Internalization problems such as anxiety and depression and problem behaviors that are manifested outwardly, such as aggressive behaviors and delinquency, are important indicators of adolescents’ adaptation [6] that require close attention. These are important in that they can bring about serious outcomes such as suicide, trigger social problems such as crimes, and can lead to maladjustment in adulthood [7].

Factors that influence adolescents’ happiness include adolescents’ self-understanding and personal psychological variables [8]. Among these factors, anger, despite the fact that it is a natural, healthy, and appropriate emotion that enhances life, can have a detrimental impact on adolescents’ psychological and physical health without appropriate management [9]. The dysfunctional aspect of anger is likely to hurt one’s own as well as others’ feelings, which would in turn destroy interpersonal relationships and lead to antisocial behaviors [10]. As anger is the direct culprit in an array of problems in adolescents [11], adolescents need to be trained to be able to understand and control their anger.

On the other hand, positive thinking, defined as an attitude that promotes positive experience of life and appropriate human growth, has a cognitive feature that creates behavioral changes, and thus can impact anger and anger management, which are influenced by the cognitive domain [12]. Further, positive thinking is positively correlated with psychological health indicators such as life satisfaction and happiness, while it is negatively correlated with psychopathological indicators such as stress, anxiety, depression, and anger [13]. Hence, it can serve as a protective factor against negative emotions or problem behaviors.

The broaden-and-build theory of positive emotions states that experience of positive emotions broadens people’s attention, thoughts, and range of actions [14], enabling individuals to become more creative, socially skilled, and healthy. Positive emotion forms psychological resource to reduce negative emotion [15], the exchange of positive emotion with other people develops positive relationship [16], and positive emotion makes positive influences through interactions with cognition, attitude, and behavior [17]. Leem et al. [18] maintained that to maximize the advantage of the intervention for positive emotion we should provide the participants with more opportunities for the experience of positive emotions because the positive emotions affect the emotion more than negative emotions. Accordingly, Kim and Lee [19] reported that the positive emotions facilitated the attempts to enforce good emotions. Gratitude among positive emotions, which is easily felt and expressed in daily living, refers to acting in appreciation of one’s internal and external environments, and consists of three properties: expression of interactions, the force that drives positive change, and phased development through training and habit formation [20]. Gratitude is strongly associated with positive emotions, happiness, life satisfaction, and social relationship satisfaction [21]. In a study on American adolescents, gratitude was identified as the most frequently reported strength associated with happiness [22], and a Korean study on adolescents also confirmed that gratitude is a significant factor in subjective wellbeing and psychological wellbeing [23].

Therefore, this study aims to develop an emotion management program for adolescents based on the broaden-and-build theory, and to evaluate its effects on subjective happiness, anger control, and gratitude to help adolescents understand and appropriately manage their emotional states such that they can positively cope with stress situations and prevent potential problems.

## 2. Purpose

The purpose of this study is to develop an emotion management program to strengthen positive emotions and evaluate the program’s effects on subjective happiness, anger control, and gratitude among high school students.

## 3. Methods

### 3.1. Research Design

This study is an experimental study using a pretest-posttest control group design.

### 3.2. Participants

The study population consisted of students of a male high school in Korea, who repeatedly underwent personal counseling with their homeroom teachers and school nurses due to problematic behaviors or were deemed in need of emotion management due to a possibility of exposure to crisis. This study was conducted on students who were vulnerable to a crisis or were in need of emotion management based on the recommendation of teachers who understood the purpose of the study. These students included those who had unexcused absences, who were involved in school violence, or who had difficult peer relationships. The inclusion criteria were as follows: high school students who consented to participate in the study, were capable of completing the questionnaire, and did not have diagnosed mental problems or other diseases. Of the 87 recruits, 74 students who met the inclusion criteria and consented to participate in the study were randomly assigned numbers, and the participants were divided into the intervention group and control group based on odd and even numbers, with 37 in each group. After excluding one participant in the control group who was withdrawn from the study for not participating in the post-program survey, a total of 37 participants in the intervention group and 36 participants in the control group were included in the final analysis.

### 3.3. Measurements

Twenty-seven items, including items for demographic characteristics, were used in this study, and instruments validated for use on adolescents were used with permission from the author.

Subjective happiness: The Subjective Happiness Scale (SHS) developed by Lyubomirsky and Lepper [24] and adapted and validated by Kim [25] was used. The SHS consists of five items to measure overall subjective happiness, and rated on a seven-point Likert scale. Positive items were summed per the quantified score, and negative items were reverse scored. The total score ranged from 5–35, with a higher score indicating a higher level of subjective happiness. The reliability of the study as measured with Cronbach’s α was 0.83 in the study by Kim [25] and 0.78 in our study.

Anger control ability: Anger control was assessed using the scale developed by Morganett [26] for use on elementary school children and modified by Gwon [27] for use on adolescents. The scale consists of 10 items, with each item rated on a five-point Likert scale. A higher score indicates more positive improvement in anger control. Cronbach’s α was 0.72 in the study by Gwon [27] and 0.78 in our study.

Gratitude: Gratitude was assessed using the Korean version of the Gratitude Questionnaire-6 (GQ-6), which was originally developed by McCullough, Emmons, and Tsang [28] and adapted into Korean by Kwon, Kim, and Lee [29]. This is a six-item scale that assessed the experiences and expressions of gratitude in daily living, with each item rated on a seven-point Likert scale. A higher score indicates a higher level of gratitude. The Cronbach’s ⍺ was 0.85 in the study by Kwon, Kim, and Lee [29] and 0.80 in our study.

### 3.4. Study Procedure

#### 3.4.1. Participant Assignment and Baseline Survey

This study was performed from May to December 2018. The control group and intervention group completed the questionnaire asking about their demographics and subjective happiness, gratitude and anger control prior to the intervention, and the data were collected by the researcher.

#### 3.4.2. Emotion Management Program

The emotion management program is a group program that was developed based on the broaden-and-build theory [14], which broadens individuals’ thoughts and ranges of actions through positive emotional experiences, and a review of literature on analysis of the construct of “gratitude” and adolescents’ emotional experiences and emotion management. The development of the program was based on the literature [30,31,32] and the data from the student interviews, and was completed by the reviews from the school nurses, mental-health nurses, and the professors in nursing schools.

For the components of the program, we referred to the strength of gratitude, which induces positive changes through a preceding factor that calms down anger and complaints and increases self-expression, as well as to the property of gratitude that enables phased development through training and habit formation [20]. We integrated the positive emotional construct of gratitude with understanding of self-existence, perception and expression of emotions, and interactions with others through presentations and feedback within the group. To enhance participants’ understanding of the program and make it more stimulating, we utilized various worksheets, videos, daily “gratitude journal” to practice emotion management skills, and a “gratitude relay” at every session. The program was designed as a five-session program. Session 1 “Orientation and Self-introduction” included an explanation about the program’s purpose and schedule, time for the participants to say hi to one another, “introducing myself”, and baseline survey. In session 2 “Self-understanding”, participants broadened their self-perception and recognized their values through “self-auction”, where they set a price for their skills, strengths, character, and dreams. In session 3 “Anger management”, participants recalled and alleviated their angry emotions and situations by watching a video about anger and forgiveness and sharing their opinions and similar cases. In session 4 “recognition and expression of gratitude”, participants explored and expressed the meaning and features of gratitude by engaging in various experiences through role play involving situations of gratitude. In session 5 “formation of habits of emotion management skills”, participants understood and practiced the effects of emotion management skills by giving a presentation about the positive effects they had experienced by applying emotion management skills and motivating themselves to fulfill their self-promised pledges at the completion ceremony.

Each session consisted of a recollection of the previous session, presentation of a gratitude journal to relax the mood, instructions about the current session, activities relevant to the topic, and wrapping up. During wrap up, participants held one another’s hands for a gratitude relay to practice positive emotional skills. After each session, feedback was provided by the session evaluation sheet. After the program, we conducted interviews with each participant. The interview questions included, “How did you think about gratitude in the past”?, and “what changes in your thoughts or behaviors about gratitude did you experience after participating in the program”? This program was administered during free study hours and not during the regular school classes, and each session lasted 50 minutes. The outline of the emotion management program is shown in Table 1.

#### 3.4.3. Pilot Study

Prior to the main program, we conducted a pilot study on 15 qualified students to assess the construction and methodology of the program. These 15 students are chosen at random from the recommended students by the teacher. Based on the results, we modified a part of the program and adjusted the duration of each session and other details.

#### 3.4.4. Post-Program Survey

We surveyed participants’ subjective happiness, anger control, and gratitude two weeks after the completion of the program in the intervention group, and the same post-program survey was conducted on the control group at the same time. The delay of two weeks was intentionally given to the participants before the post-program survey to allow for their gratitude habits to form after the program. The collected data were processed by assigning a number for each participant per the guidelines for the processing of personal information, and the participants were given a small gift at data collection.

### 3.5. Analysis

The collected data were analyzed using SPSS 25.0 software (IBM, Armonk, NY, USA). Demographic characteristics were presented as percentages, and the homogeneity of demographic characteristics between the intervention group and control group was tested with Fisher’s exact test and Chi-square test. Normal distribution of the study variables gratitude, subjective happiness, and anger control was tested with the Shapiro-Wilk test. Homogeneity of the study variables between the two groups was analyzed with an independent *t*-test and the Mann-Whitney U test. To analyze the effects of the intervention program, normally distributed gratitude and subjective happiness were analyzed with an independent *t*-test and non-normally distributed anger control was analyzed with the Mann-Whitney U test. Statistical significance was set at 0.05.

### 3.6. Ethical Consideration

This study was approved by the institutional review board of Dongyang University (1041495-201703-HR-04-01) and was performed from May to December, 2018. We adequately informed the participants of the purpose of the study, study procedure and contents, use of personal information, benefits and drawbacks anticipated by study participation, confidentiality, and ability to withdraw from the study at any time, and obtained written informed consent.

## 4. Results

### 4.1. Homogeneity of Demographic Characteristics Between Two Groups

#### 4.1.1. Homogeneity Test of Demographic Characteristics Between Two Groups

There were no significant differences in age, living with parents, living with grandparents, siblings, economic status, and religion between the intervention and control groups, confirming the homogeneity of the two groups (Table 2).

#### 4.1.2. Homogeneity Test of Dependent Variables Between Two Groups

There were no significant differences in subjective happiness, anger control, and gratitude between the two groups before the program (Table 3).

### 4.2. Effects of the Emotion Management Program

There was a significant difference in the mean subjective happiness score between the two groups (t = 3.409, *p* = 0.001). The mean subjective happiness score increased by 0.65 after the intervention in the intervention group while it decreased by 0.08 in the control group, showing a significant difference (Table 4).

There was no significant difference in the mean anger control score between the two groups (t = 0.298, *p* = 0.767). The mean anger control score decreased by 0.00 in the intervention group and by 0.03 in the control group, with no significant difference between the two groups (Table 4).

There was no significant difference in the mean gratitude score between the two groups (z = 0.528, *p* = 0.599). The mean gratitude score increased by 0.44 in the intervention group and by 0.31 in the control group, with no significant difference between the two groups (Table 4).

## 5. Discussion

This study aimed to develop an emotion management program and examine the effects of the program on subjective happiness, anger control, and gratitude in high school students, who are experiencing adolescence and an array of changes.

Subjective happiness increased in the intervention group after the intervention compared to the baseline, showing a statistically significant difference between the intervention group and control group. This can be interpreted in the same context as the greater effects of a group program on happiness compared to positive emotions, psychological wellbeing, self-respect, and depression in the study by Yoo and Son [33]. Subjective happiness should be understood in consideration of various environmental factors related to adolescents’ growth and development. As adolescents are at an emotionally unstable stage of growth, and they spend most of their time with peers in and outside their schools, interaction with friends may have an impact on adolescents’ emotions. Particularly, positive relationships with close same-sex friends are an essential component of adolescents’ happiness [34].

The intervention group performed activities involving recognition and expression of positive emotions with peers. After the end of the program, the participants stated that expressing gratitude to friends was amazing and impressive and that they became closer with their friends by verbally thanking them once a day. Moreover, they were able to positively understand themselves and discover their values through activities such as coming up with desired nicknames and setting a price for their strengths, weaknesses, hidden talents, and dreams. Discovering the value of themselves as well as others and adopting a respectful attitude through the presentations and sharing their learning with the participants is speculated to have contributed to boosting their subjective happiness. Further, we can surmise that the program contributed to alleviating their tension from excessive academic stress caused by regular coursework and additional learnings, and inducing catharsis.

The emotion management program developed in this study was designed to help participants recognize and express their emotions and practice positive emotional skills using the properties of gratitude, and is similar to Thompson’s emotional self-regulation [35]. The goal of this program was to improve the participants’ thoughts and behaviors through positive emotional experiences. Despite the fact that the intervention group reported expressing gratitude to friends in this program as impressive and showed a significant elevation of subjective happiness, there were no significant differences in the level of gratitude between the two groups. These results call for an examination of whether the number of sessions in the program was appropriate for increasing gratitude and whether participant preparation and program methodology were appropriate.

Gratitude refers to a generalized tendency of recognizing others’ contributions to positive experiences and outcomes and responding with a grateful heart [28], and it determines the threshold at which a particular form of emotional state occurs [36]. Experiencing and expressing gratitude in adolescence, during which individuals must form their identities and social relationships, not only enhances positive emotions and strengthens social bonding [37] but also improves academic performance and increases satisfaction with school life by boosting students’ motive for accomplishment [38] and increases subjective happiness [36]. The intervention group wrote three things they feel thankful for every day during the program and participated in a gratitude relay, where they expressed gratitude to others. After participating in the program, the intervention group stated that they “learned how to feel grateful for the little things in their lives, express gratitude more frequently, and perceiving stress positively”. In this study, the level of change of gratitude in the intervention group did not significantly differ from that of the control group. This result contrasts with a prior study [39], which asked the participants, adult females, to write appreciation journal every day for two weeks to improve gratitude disposition. The significance of this study, however, lies in its positive messages about gratitude. Gratitude is an expression of interaction, and people can develop gratitude in phases through training and habit formation [20]. The training and habit formation of our program seems to be less effective because of the lack of thorough examination of the individual’s emotions. Therefore, replication studies examining the optimal duration, timing, and approach of the program are needed. In particular, because of the varying contents and types of gratitude depending on the physical, emotional, and environmental factors facing adolescents, individualized interventions should also be considered.

Our results showed that there was no significant difference in anger control between the intervention group and control group. This is consistent with the results of Lee and Yang [40] but contradictory to the findings of Lök, Bedemli, and Canbaz [9], where anger management education positively reduced the interpersonal relational response to anger and some factors of anger-related behaviors. Anger is a part of an oppressed internalized emotion, as opposed to an emotion expressed at the conscious level, and how well an individual controls their anger in an anger-provoking situation is important [40]. Anger control is not simply a result of changed perceptions about the emotion of anger at the instant, and it can be gradually improved through correction of the automatic thoughts and negative perceptions related to anger and by learning coping skills. In other words, controlling and managing anger helps individuals to sedate, suppress, and control anger-provoking negative emotions [41].

In our program, anger management was dealt only in one session, so it is possible that the number of sessions directly dealing with anger was not enough for students to recognize and control their anger. This is evident in the post-program statements of the intervention group, “At first, I was so embarrassed to talk about myself in front of other people and I was frustrated because I didn’t know what to say”. We can speculate that adolescents might have had difficulty concentrating in the anger management session due to the feeling of awkwardness they had during activities encouraging them to verbally and behaviorally express their emotions. Therefore, a sufficient number of sessions should be allocated for anger exploration and management in the future. Additionally, considering that adolescents feel awkward expressing themselves, phased and individualized approaches are needed to help them naturally recognize and control anger.

Candelaria, Fedewa, and Ahn [42] reported in their meta-analysis of studies on anger management interventions that anger management interventions teach adolescents coping skills and that emotional recognition, relaxation techniques, a problem-solving cognitive behavioral approach, and coping skill training are successful. Anger control is known to be influenced by adolescents themselves and their families as opposed to their school environment [43]. This suggests that adolescents should accurately recognize their emotions and strive to mitigate negative emotions and convert them into positive emotions and that family bonding and support is more important than anything else. Thus, it would be desirable to allocate sufficient time in the program such that adolescents can recognize their emotions and to devise appropriate intervention strategies depending on the level of emotional risk. Furthermore, adequate awareness of anger and parental education for anger control and management should be given to enable positive family dynamics. Most of all, school healthcare personnel must gain a thorough understanding of adolescents’ anger experiences, and families and communities should work in collaboration to regulate and manage them.

## 6. Limitation

The results obtained from this study cannot be generalized because the subjects of the study were the students recommended for the emotional intervention program by a single High School. In addition, the different reasons for each recommendation was not considered in the program design. The study has limitations because the intervention program was provided without thoroughly assessing the individual psychological status of the participating students. The sample size was computed using the G-power 3.1 program. For a comparison of means between two groups, the required sample size for an α of 0.05, power of 0.80, and a medium effect size d of 0.80 was 64 for the intervention group and 64 for the control group. The choices of α, power, and medium effect size are based on Moltrecht et al. [44], which justified the moderate effect size for emotion regulation according to the result of meta-analysis on the 21 psychological intervention research for improving emotion control of adolescents. The subject size of this study was 37 for experimental group and 36 for control group, which were less than the recommended size. To evaluate the appropriateness of the sample size, Cohen’s [45] power analysis was performed on the subjective happiness, which showed significant differences between the experimental and control group, and the effect size was 0.78. The test power of a post-hoc test with significance level of 0.05 was computed as 0.90, justifying the appropriateness of the sample size and effect size of this study. When we performed a priori power analysis with effect size of 0.99, the necessary sample size for each group was 18 or more.

## 7. Practical Implications

Based on our results, it would be more effective to develop emotion management programs based on appropriate peer interactions, and, as an array of variables influence adolescents’ emotions, these programs should adequately consider personal and environmental factors. Considering that emotions in adolescence motivate behavior and that positive emotions and happiness experienced during growth and development can have a persistent impact on their future lives, emotion management programs for adolescents should be designed as a continuous and linked program, as opposed to a one-time program, and education for school teachers is crucial to properly manage adolescents before problem behaviors surface. Moreover, emotion management should be naturally trained through the school curriculum to help adolescents maintain and promote healthy psychological emotions, and, to enable effective implementation in the curriculum, various strategies should be explored and the programs should be linked to and run in collaboration with local communities.

## 8. Conclusions

This study aimed to develop and validate the effects of an emotion management program to help male late high-school adolescents with their daily lives and school adaptation. The results showed that the intervention had a significant elevation of subjective happiness, but no significant influence on their gratitude and anger control. This result suggests that the emotion management program was partially effective.

This study also suggests the need for further research that utilizes various personal, friend, school, and community-related variables to produce positive effects and sophisticated study designs that can contribute to improving both gratitude and anger control. We also suggest to include more number of participants in the study to satisfy the statistical requirements for all the variables under investigation.

## Figures and Tables

**Table 1:** Emotion management program for late adolescent male students.

Session		Topic	Content
1	Orientation And self-introduction	Program orientation and pledge ceremony	-Orientation, instructions about writing a gratitude journal -Pledge ceremony -Baseline survey (pre-test)
		Introducing “myself”	-Come up with desired nicknames -Introduce yourself
2	Self-understanding	Knowing your values	-Share of thankyou journal -Set a price for your strengths, character, and dream -Raise your values
3	Anger management	Looking into your hurt feelings (Understanding anger)	-Share of thankyou journal -Recall a moment of anger -Share the automatic thoughts that you had when you were hurt -Watch a video about anger (role play) and share your experiences
4	Recognition and expression of gratitude	Knowing gratitude	-Share of thankyou journal -Share your thoughts and feelings about gratitude -Share your experiences of gratitude -Write a list of grateful things
5	Formation of habits of emotional management skills	Expressing gratitude Forming habits of gratitude	-Share of thankyou journal -Share the expressions of gratitude -Thank yourself, thank others -Expressions of gratitude by level -Practice expressions of gratitude through a role play
			-Share the changes you had after the gratitude program -Write a letter of gratitude -Rolling paper
		Completion ceremony	-Thank You Friends completion ceremony

**Table 2:** Homogeneity test of demographic characteristics between the two groups.

Characteristics	Categories	Exp. (n = 37)	Cont. (n = 36)	t/*x*^2^ (*p*)
		n (%)	n (%)	
Age (years)	(M ± SD)	17.21 ± 0.75	16.91 ± 0.73	1.726(0.089)
Live with parents	Yes	34(91.9)	34(94.4)	0.186(0.666)
	No	3(8.1)	2(5.6)	
Live with grandparents	Yes	4(10.8)	7(19.4)	1.063(0.303)
	No	33(89.2)	29(80.6)	
Have siblings	Yes	10(27.0)	5(13.9)	1.929(0.165)
	No	27(73.0)	31(86.1)	
Economic status	High	11(29.7)	8(22.2)	0.574(0.750)
	Middle	21(58.8)	22(61.1)	
	Low	5(13.5)	6(16.7)	
Religion	Yes	27(73.0)	31(86.1)	1.929(0.165)
	No	10(27.0)	5(13.9)	

Exp. = Experimental group; Cont. = Control group.

**Table 3:** Homogeneity test of dependent variables between the two groups.

	Range	Exp. (n = 37)	Cont. (n = 36)	*x*^2^ (*p*)
		M ± SD	M ± SD	
Subjective happiness	1~7	4.84 ± 0.97	4.79 ± 0.75	0.238(0.812)
Anger control ability	1~5	3.60 ± 0.55	3.37 ± 0.59	1.728(0.088)
Gratitude	1~7	5.00 ± 0.82	4.63 ± 0.88	1.839(0.070)

Exp. = Experimental group; Cont. = Control group; M = Mean; SD = standard deviation.

**Table 4:** Comparison of subjective happiness, gratitude, and anger control ability between two groups.

Variable		Exp. (n = 37)	Cont. (n = 36)	t/z (*p*)
		M ± SD	M ± SD	
Subjective happiness	Pre-test	4.84 ± 0.97	4.79 ± 0.75	0.238(0.812)
	Post-test	5.50 ± 0.85	4.70 ± 0.66	4.435(< 0.001)
	Difference	0.65 ± 1.10	−0.08 ± 0.73	3.409(0.001)
Anger control ability †	Pre-test	3.60 ± 0.55	3.37 ± 0.59	1.728(0.088)
	Post-test	3.61 ± 0.60	3.33 ± 0.51	2.084(0.041)
	Difference	−0.00 ± 0.62	−0.03 ± 0.55	0.332(0.740)
Gratitude	Pre-test	5.00 ± 0.82	4.63 ± 0.88	1.839(0.070)
	Post-test	5.45 ± 1.06	4.95 ± 0.94	2.134(0.036)
	Difference	0.44 ± 1.21	0.31 ± 0.86	0.528(0.599)

Exp. = Experimental group; Cont. = Control group; M = Mean; SD = standard deviation. † Mann-Whitney U test.
